# Cost and community acceptability of enhanced antibiotic distribution approaches for trachoma in the Republic of South Sudan: enhancing the A in SAFE (ETAS) study protocol

**DOI:** 10.1186/s12886-023-02783-x

**Published:** 2023-02-06

**Authors:** Angelia M. Sanders, Samuel Makoy, Andrew R. Deathe, Stephen Ohidor, Timothy C. Jesudason, Andrew W. Nute, Patrick Odongi, Lochebe Boniface, Stella Abuba, Alexis S. Delahaut, Wilson Sebit, James Niquette, E. Kelly Callahan, Damian G. Walker, Scott D. Nash

**Affiliations:** 1 The Carter Center, Atlanta, GA USA; 2 Ministry of Health, Juba, Republic of South Sudan; 3 The Carter Center, Juba, Republic of South Sudan; 4 Partners in Global Health Ltd, Dereham, UK; 5 Independent Contractor, Arlington, VA USA

**Keywords:** Trachoma, Mass drug administration, South Sudan

## Abstract

**Background:**

The World Health Organization targeted trachoma for global elimination as a public health problem by 2030. Reaching elimination thresholds by the year 2030 in the Republic of South Sudan will be a considerable challenge, as the country currently has many counties considered hyper-endemic (> 30% trachomatous inflammation-follicular [TF]) that have yet to receive interventions. Evidence from randomized trials, modeling, and population-based surveys suggests that enhancements may be needed to the standard-of-care annual mass drug administration (MDA) to reach elimination thresholds in a timely manner within highly endemic areas. We describe a protocol for a study to determine the cost and community acceptability of enhanced antibiotic strategies for trachoma in South Sudan.

**Methods:**

The Enhancing the A in SAFE (ETAS) study is a community randomized intervention costing and community acceptability study. Following a population-based trachoma prevalence survey in 1 county, 30 communities will be randomized 1:1 to receive 1 of 2 enhanced MDA interventions, with the remaining communities receiving standard-of-care annual MDA. The first intervention strategy will consist of a community-wide MDA followed by 2 rounds of targeted treatment to children ages 6 months to 9 years, 2 weeks and 4 weeks after the community MDA. The second strategy will consist of a community-wide biannual MDA approximately 6 to 8 months apart. The costing analysis will use a payer perspective and identify the total cost of the enhanced interventions and annual MDA. Community acceptability will be assessed through MDA coverage monitoring and mixed-methods research involving community stakeholders. A second trachoma-specific survey will be conducted 12 months following the original survey.

**Discussion:**

ETAS has received ethical clearance and is expected to be conducted between 2022 and 2023. Results will be shared through subsequent manuscripts. The study’s results will provide information to trachoma programs on whether enhanced interventions are affordable and acceptable to communities. These results will further help in the design of future trachoma-specific antibiotic efficacy trials. Enhanced MDA approaches could help countries recover from delays caused by conflict or humanitarian emergencies and could also assist countries such as South Sudan in reaching trachoma elimination as a public health problem by 2030.

**Trial registration:**

This trial was registered on December 1^st^, 2022 (clinicaltrails.org: NCT05634759).

**Supplementary Information:**

The online version contains supplementary material available at 10.1186/s12886-023-02783-x.

## Background

The World Health Organization (WHO) has targeted trachoma for global elimination as a public health problem by 2030 (trachomatous inflammation-follicular [TF] < 5% among children ages 1 to 9 years; and trachomatous trichiasis [TT] < 0.2% in those 15 years and older) [1]. Although progress has been made in reducing the burden of trachoma in many endemic countries, some countries are experiencing persistently endemic trachoma after many years of interventions, and countries affected by conflict and humanitarian emergencies could take decades to reach trachoma elimination targets [2, 3]. The “A” component of the WHO-endorsed SAFE (surgery, antibiotics, facial cleanliness, and environmental improvement) strategy focuses on mass drug administration (MDA), particularly with azithromycin, targeting all members of an endemic district where the prevalence of active trachoma is ≥ 5% among children ages 1 to 9 years.

Evidence for the importance of enhanced MDA strategies is growing, particularly with the help of modeling studies [4, 5]. Recent work by the Neglected Tropical Disease (NTD) Modelling Consortium has demonstrated that in some hyperendemic districts and countries, standard-of-care annual community-wide MDA will not result in a district reaching the elimination of trachoma as a public health problem threshold within 10 years [5]. Fortunately, models have projected that alternative enhanced MDA strategies can change the annual trajectory of trachoma prevalence [5]. They have suggested that 1 alternative strategy, characterized by a community-wide MDA followed by 2 extra rounds of treatment to children ages ≤ 9 years, could lead to elimination faster compared to standard-of-care annual MDA. Empirical data from endemic communities under different alternative MDA strategies are needed to verify these modeling results.

The Republic of South Sudan has historically had one of the highest burdens of trachoma in the world [6, 7]. While the South Sudan Ministry of Health (MOH) has been implementing the SAFE strategy in various counties (the equivalent of districts) throughout the country for over 10 years, there remain many counties considered to be hyper-endemic (> 30% TF). Furthermore, a considerable proportion of the country has not yet been assessed for trachoma prevalence, and thus interventions have not yet started. Eliminating trachoma as a public health problem by the year 2030 in South Sudan will be a considerable challenge [7].

This proposed study will estimate the cost and community acceptability of enhanced MDA strategies in South Sudan, which along with the trachoma survey data from this study will be important for the future planning and design of comparative efficacy trials. If efficacious and cost-effective, enhanced MDA approaches could help countries recover from delays in programming caused by conflict, humanitarian emergencies, or COVID-19; and could also assist countries, including South Sudan, in reaching trachoma elimination as a public health problem within years instead of decades [5]. This has the potential to not only reduce disease burden precipitously but also could result in significant cost savings over the long term to countries and donors.

## Methods

### Study design overview

**Fig. 1:**
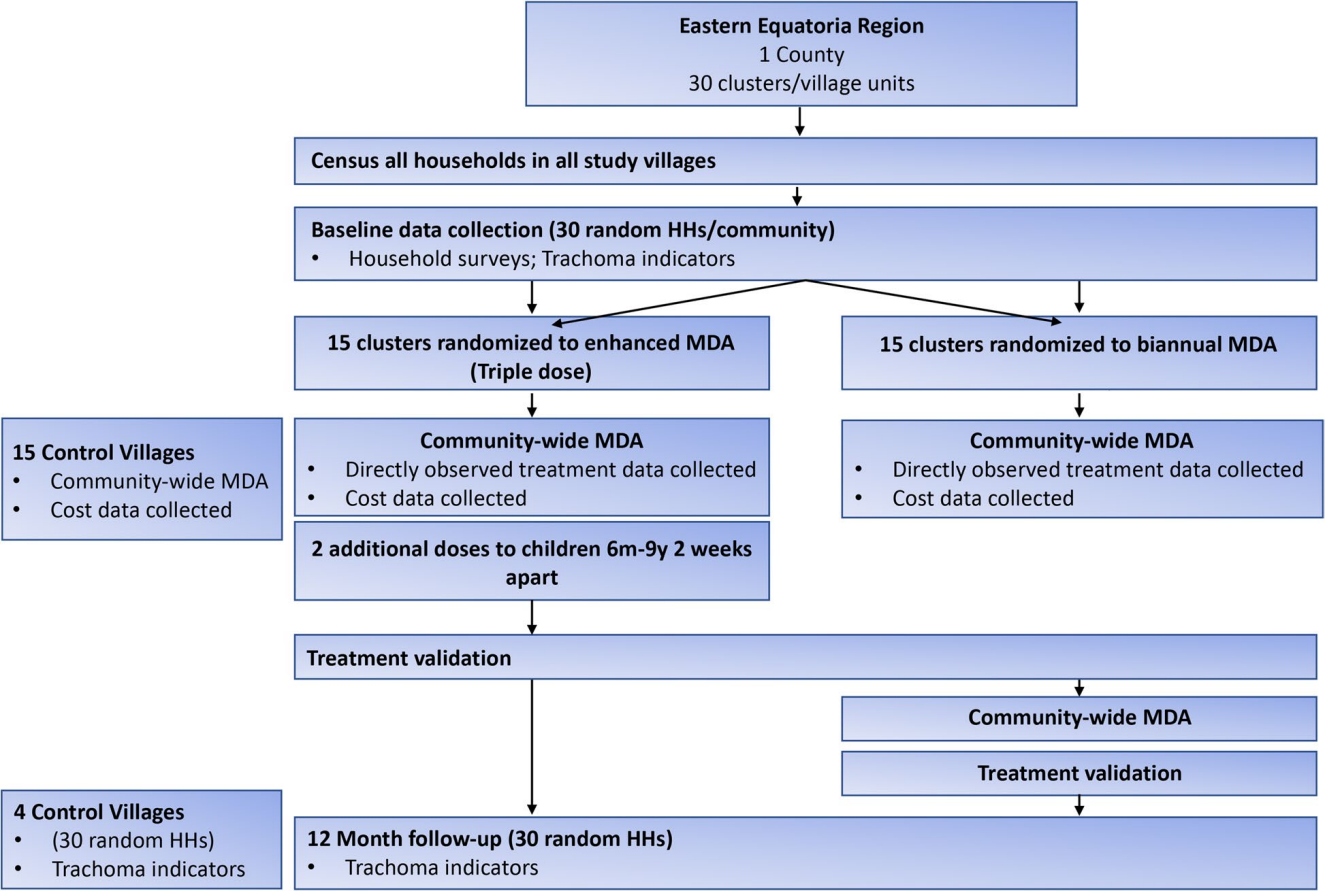
ETAS study design summary for communities included in the primary outcome analysis. HH = Household; MDA = Mass Drug Administration

The Enhancing the A in SAFE (ETAS) study is a community randomized study to evaluate cost and community acceptability of enhanced antibiotic trachoma interventions within an endemic county in South Sudan. A population-based prevalence survey will be conducted whereby 30 communities are selected for trachoma assessment. Following the survey, communities will be randomized 1:1 to receive 1 of 2 enhanced antibiotic interventions (Fig. 1). The remaining communities in the county will receive standard-of-care annual MDA. The primary outcomes of the study are the cost of enhanced MDA compared to standard-of-care annual MDA and the community acceptability of enhanced MDA within study communities. A trachoma impact survey will be conducted approximately 12 months following the original trachoma prevalence survey. The total study duration will be approximately 13 months from the start of the community-level census to the time of the 12 month follow up impact survey. This protocol adheres to the Standard Protocol Items: Recommendations for Interventional Trials (SPIRIT) guidelines and refers to version 1.2 of the study protocol as revised 5 May 2021.

### Setting, eligibility, and participants

**Fig. 2:**
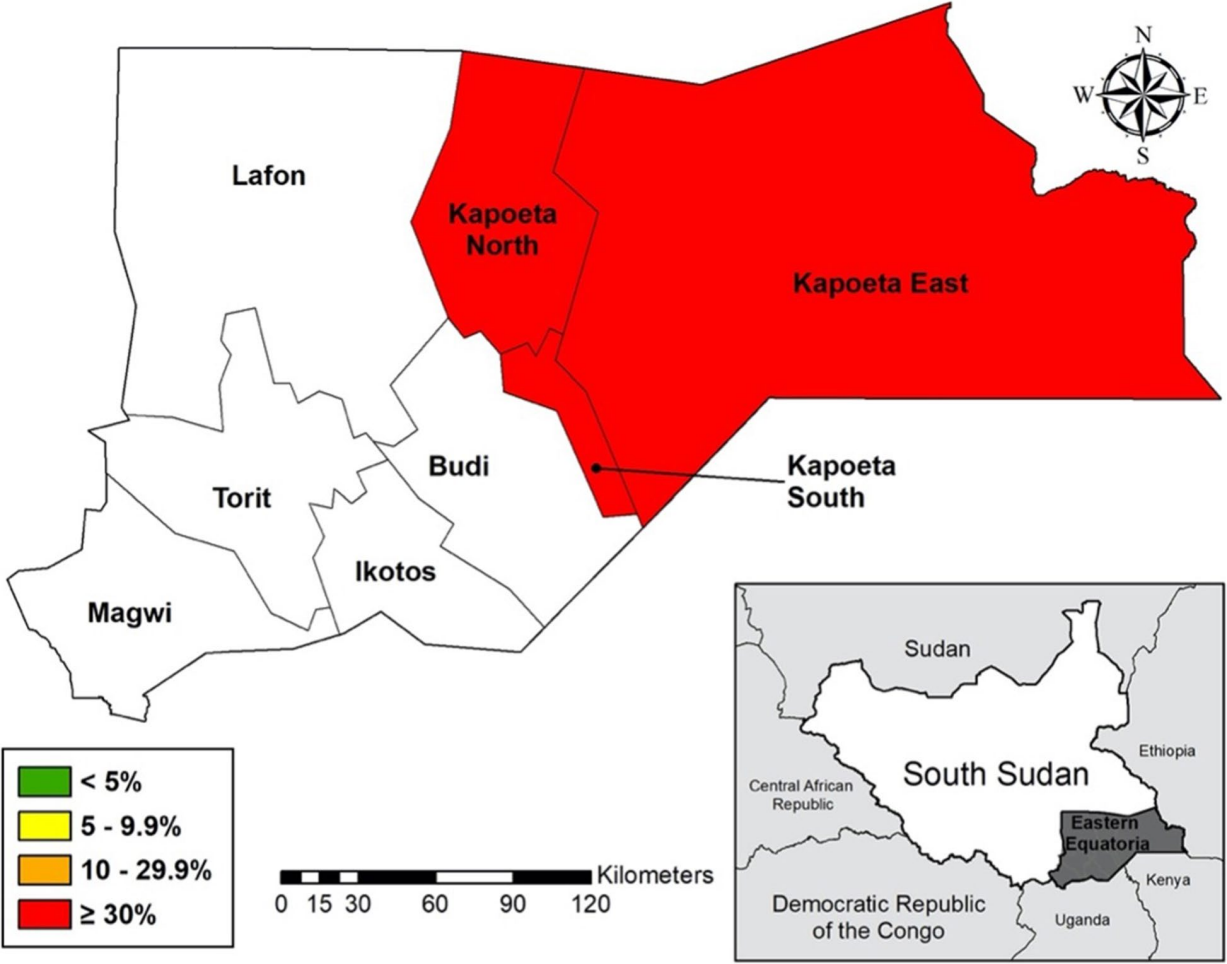
ETAS study area in Eastern Equatoria state, South Sudan with 2015 TF prevalence estimates shown

The study will be conducted within a trachoma endemic county in Eastern Equatoria state, South Sudan (Fig. 2). This area was chosen based on previous survey data demonstrating a high burden of TF among children ages 1 to 9 years ranging from 30 to 48% [7]. The baseline for this study will be timed to coincide with the scheduled programmatic trachoma impact surveys in Eastern Equatoria state. For purposes of this manuscript, the initial survey connected to this study will be referred to as a baseline survey and the survey conducted at completion of the study will be referred to as an impact survey (survey methods described below). As part of the baseline survey to determine TF prevalence in the study county, 30 communities will be randomly selected from a geographically ordered list provided by the South Sudan MOH Trachoma Control Program [8]. All 30 communities will be enrolled in this randomized community study. Communities are eligible to participate if they are in the study county regardless of the individual-community level TF prevalence. There are no exclusion criteria for community-level participation. Within study communities, all households and individuals will be eligible for participation. Children ages 6 months to 1 year will be eligible for azithromycin interventions in this study as they are normally targeted with this medication as part of programmatic interventions and in previous randomized trials, they could potentially serve as an infection reservoir if not treated and are an age group which may benefit from the ancillary effects of azithromycin on mortality [9–13]. Because of the observed mortality benefit to these young children, ongoing studies are now randomizing children in this age group to multiple doses of azithromycin per year at national levels [14].

For the 30 communities enrolled in the randomized community study, we expect an average population per community (based on programmatic data) of 649 individuals. This would lead to MDA treatment data on approximately 19,470 individuals of all ages. Since children ages 6 months to 9 years account for approximately 40% of the population, we expect to have MDA treatment data on 260 children per cluster or 7,788 total children in this age range.

### Interventions

Antibiotic interventions will take place at the community level. The first intervention strategy will consist of an enhanced antibiotic regimen comprised of a community-wide MDA, followed by 2 additional rounds of targeted treatment to children ages 6 months to 9 years. The targeted treatments will be distributed 2 weeks and 4 weeks after the community-wide treatment, resulting in 3 doses to children 6 months to 9 years. The second strategy will consist of a community-wide biannual MDA approximately 6 to 8 months apart, resulting in 2 doses for the targeted community. Timing of the second dose will be based on access to communities and at least 6 months after the first dose.

Azithromycin, also known in the global trachoma program by the brand name Zithromax®, is a macrolide antibiotic and is used in the treatment of active trachoma. Zithromax® is donated to the South Sudan MOH for MDA by Pfizer Inc., through the International Trachoma Initiative (ITI). The recommended dosage for the treatment of trachoma is a single dose of 20 mg/kg of body weight and is implemented using an “age-height” based dosing strategy for the trachoma MDA campaigns. Azithromycin can be administrated in either tablet form or powder for oral suspension (POS). Dosing and administration for this study will be conducted following procedures outlined in the Zithromax® Management Guide [15]. To reduce the risk of choking, all children participating in the study intervention ages 6 months to 9 years will receive azithromycin by POS only. Populations ineligible for azithromycin based on national guidelines, including infants under 6 months of age, pregnant women in their first trimester, individuals with a previous history of severe adverse events (SAEs), and individuals with severe illness at the time of MDA, will be offered treatment with 1% tetracycline eye ointment (TEO), to be applied twice daily to both eyes for a 6-week period [15]. Trained drug distributors will facilitate drug administration for the appropriate treatment dose by assessing everyone that presents. Those receiving antibiotic will be directly observed by the distribution team.

Throughout this study, existing S, F, and E interventions will continue through standard programming. This includes offering surgery to individuals identified as needing TT surgery, and advocacy work with water, sanitation, and hygiene (WASH) focused non-governmental organizations operating in the country.

### Adverse events

Azithromycin is generally well tolerated and adverse events are rare [16–18]. The most commonly reported side effects of azithromycin are gastrointestinal effects which include diarrhea, nausea, abdominal pain, and vomiting. As part of MDA training, all drug distributors, community chiefs, and government supervisors are trained on side effects and how to respond to SAEs. All study participants will be informed of potential side effects of azithromycin and screened for any history of SAEs from azithromycin. All drug recipients will be informed to immediately report to the drug distributors or community chief if they experience signs and symptoms of any SAE. All suspected SAEs will be recorded, immediately managed via the nearest health facility, investigated, and reported according to South Sudan MOH guidelines and will be recorded using an SAE reporting form. SAEs will be reported to The Carter Center, Pfizer Inc., and the Institutional Review Boards (IRB) within 24 h. An SAE officer will be stationed within the study area for the duration of the project to monitor reports and assist key government staff in investigating and facilitating rapid responses to suspected SAEs during the intervention period. While this study will include increased intensity of antibiotic pressure, Chlamydia-specific antimicrobial resistance to azithromycin has yet to be demonstrated as part of trachoma MDAs, including those with multiple rounds per year [19].

### Treatment masking

Because of the community nature of the MDA treatments, masking will not be possible in this study. However, MDA treatment teams will be different from teams collecting trachoma indicator data. Laboratory and photographic grading team members will be masked to the treatment arm of the study.

### Intervention costing

The costing analysis will determine the relative costs of the enhanced MDA strategies, including the triple dose and biannual MDA, which will be compared to each other, and compared to the standard-of-care annual MDA. This study will utilize the Global Health Cost Consortium Reference Case for Estimating the Costs of Global Health Services and Interventions and the Global Health Cost Consortium Principles and Methods Reporting Checklist to ensure the quality and comparability of results [20, 21]. The costing analysis will provide the evidence base to inform resource allocation decisions by the South Sudan MOH and partners within the country.

The study will take a payer perspective and encompass the costs incurred by The Carter Center as the technical and financial supporting implementing partner. This will be used to identify the cost per person treated and total costs associated with each arm of the study and annual MDA. To reduce the potential for bias, cost data will be collected using the same methods from 15 randomly selected communities in the study county receiving the standard-of-care annual MDA. Taking an ingredients-based approach, the study will include a detailed micro-costing database that captures all financial and economic costs incurred. The study will use the community level as the unit of analysis for total costs and calculate the unit cost per person treated using a top-down approach, dividing the total cost by the number of people treated.

The quantity and price of all required resources will be identified to evaluate total intervention costs for each arm of the study as well as the standard-of-care annual MDA (Table 1). To ensure costs such as salaried labor are not double counted, some activities that include staff time, including social mobilization and training, will not be broken down into all specific components. Salaried costs, for example, will be included under central administration and not included in each cost category. Where possible, data will be extracted from The Carter Center and South Sudan MOH databases.

**Table 1:** Summary of activity costs included in each cost category within the ETAS study

Input/line item	Planning	Training	Community sensitisation	Drug transportation	Drug administration	Supervision	Surveillance	Reporting	Central administration
*Capital Costs*									
Buildings	x	x	x	x	x	x	x	x	x
Communication and IT equipment	x	x	x	x	x	x	x	x	x
Accommodation equipment		x	x		x	x	x		
Vehicles	x	x	x	x	x	x	x	x	x
Other capital	x	x	x	x	x	x	x	x	x
*Recurrent costs*									
Salaried labor	x	x	x	x	x	x	x	x	x
Volunteer labor		x	x		x		x		
Accommodation	x	x	x	x	x	x	x	x	x
Per diem and travel allowances		x	x	x	x	x	x		
Zithromax					x				
Powder oral suspension					x				
Tetracycline eye ointment					x				
Utilities	x	x	x	x	x	x	x	x	x
Personal Protective Equipment	x	x	x	x	x	x	x	x	x
Security services	x	x	x	x	x	x	x	x	x
Communication	x	x	x	x	x	x	x	x	x
Transport/fuel	x	x	x	x	x	x	x	x	x
Vehicle maintenance	x	x	x	x	x	x	x	x	x
Printing	x	x	x	x	x	x	x		x
Laboratory equipment							x		
Other recurrent	x	x	x	x	x	x	x	x	x

Real world costs will be presented in 2 ways: 1) costs related to the programmatic implementation setting (research activities excluded) and 2) costs related to the study setting (including study and implementation costs) (Supplemental Table S1). Study related costs will be helpful in planning future MDA trials within this setting. Cost categories will include capital costs and recurrent costs. Cost activities will include planning, training, community sensitization, drug transportation, drug administration, supervision, surveillance, reporting, and central administration.

The economic cost of capital items, i.e. those only incurred once and with a value of 100 USD or more, will be annualized based on a 3% discount rate and estimates of the useful life of the item [22]. A “useful life” is defined as the period during which an asset or property is expected to be usable for the purpose it was acquired. For vehicles specifically, we will calculate the daily cost of a vehicle in relation to its useful life and discount rate. The financial costs of capital items will be estimated by dividing by the number of years of useful life without discounting (straight line depreciation). Rental costs will be used to determine the cost of office buildings. If a building is owned, the rental cost will be used as a cost indicator. Recurrent costs will be disaggregated from The Carter Center budget and assigned to the cost data categories for each treatment arm after each intervention phase as well as the standard-of-care annual MDA. Personnel costs will include participation in the intervention (e.g., per diems), as well as the relevant share of that person’s salary and fringe benefits that cover their working time as part of the intervention [23]. The proportion of salaries and benefits that are attributable to MDA activities will be estimated by program staff.

Commodities include the study drug, Zithromax®, which is donated, as well as TEO, which is purchased by The Carter Center. The cost of TEO will be included as a financial and economic cost. The economic costs of donated medicines will also be calculated. List costs will be derived from the International Medical Products Price Guide [24].

To estimate the costs associated with a programmatic setting, study and research costs will be removed to make costs comparable to the control communities. These costs would be those associated with increased supervision and monitoring, increased training for data collection, data collection, and administration of the study, and they will be separated among other costs. Further analysis will use the costs associated with the 2 intervention delivery methods and will estimate the potential costs incurred if each intervention was scaled up region- or country-wide. This will be calculated by multiplying the unit cost per person treated by the number of people requiring antibiotics, as stated in the WHO Alliance for the Global Elimination of Trachoma by the year 2020 (GET2020) database [25].

All costs will be presented in constant 2022 USD. If required, costs will be first converted from the local currency to USD, using the World Bank exchange rate relating to the time period during which the cost data were collected. The values will then be inflated using US inflation rates to the base year of the analysis [26, 27].

#### Economies of scale

Costing analyses of MDA programs often assume an intervention’s cost per treatment is constant, regardless of the number of individuals treated. This is a misleading way to consider the delivery costs of MDA, due to the occurrence of economies/diseconomies of scale [28]. This analysis will explore the effect of economies of scale by presenting the cost per person treated at 3 different coverage levels (60%, 80%, 90%).

#### Sensitivity analysis

Univariate and multivariate sensitivity analysis will be conducted to identify main cost drivers and identify a range of potential program costs. The sensitivity analysis will explore changes to the cost of drugs, treatment delivery, security, and per diems, and assumptions around the useful life of capital items. Costs of personal protective equipment associated with COVID-19 prevention will be removed in the sensitivity analysis. The sensitivity of the discount rate will also be varied from 0–6%.

### Community acceptability

#### Antibiotic coverage

Prior to the MDA interventions, a household census will be conducted in all 30 communities whereby all members of all households will be enumerated and the name, sex, and age of everyone will be recorded via electronic census forms. A household member is defined as any person who has lived for at least one month in the household and eats and sleeps in the household. Global positioning system (GPS) coordinates will be captured for each household. The study community census will be used to establish the population to be targeted for study interventions and will provide the denominator for MDA coverage calculations. New residents migrating into study areas after the census but prior to MDA treatment will be added to the census as they are detected. Further, residents unknown to the study team during the MDA treatment will also be added to the census and subsequently treated, if eligible.

During the MDA, each administered dose will be recorded into the electronic record created during the community census by a trained data collector who travels with the distribution team. This record will be used for both study arms to determine the antibiotic coverage during each of the subsequent treatment rounds, allowing for the calculation of the number of doses each participant received. The definition of coverage for this study is: the number of individuals ages 6 months to 9 years who received the drug in study communities in each treatment round divided by the number of children ages 6 months to 9 years enumerated in the baseline census. A secondary analysis will calculate the antibiotic coverage in each round for the age groups 10 to 15 years and ≥ 15 years. Further analyses on compliance will examine the extent and predictors of missed rounds among the 2 study arms.

The dataset on treatment coverage will be compiled quickly by the study coordinator and will be used to monitor the success of the intervention. If the coverage is lower than expected, < 85% among children ages 6 months to 9 years, the treatment team will be redeployed to conduct a community mop-up to improve treatment coverage.

While MDA treatment coverage will be monitored using the census and treatment records, treatment validation will also be conducted. Validation will be based on the WHO endorsed Supervisor’s Coverage Tool [29]. After each round of treatment (either community-wide or targeted) a random selection of 5 study communities will be chosen for treatment validation. A new team will return to a random sample of 20 households in each selected community to assess self-reported treatment within 1 week of treatment. Self-reported coverage from the modified Supervisor’s Coverage Tool will then be compared to the coverage reported in the treatment register.

#### Community awareness and acceptability

**Fig. 3:**
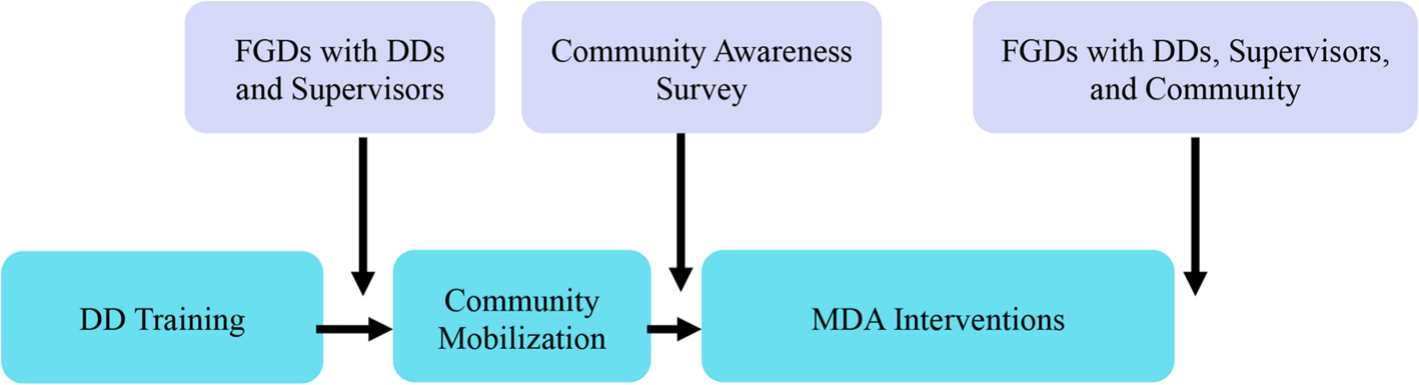
Timing of mixed methods study collection as part of the ETAS study. DD = Drug Distributor; FGD = Focus Group Discussion; MDA = Mass Drug Administration

A mixed methods approach, including focus group discussions (FGD) and community awareness surveys, will be used to elucidate insights from several groups of key community members on the acceptability and feasibility of enhanced MDA strategies and of targeting children for interventions (Fig. 3).

Three thematic areas will be the focus of the FGDs: 1) Pre-MDA FGDs with drug distributors and MDA field supervisors; 2) Post-MDA FGDs with participating drug distributors; and 3) Post-MDA FGDs with participating community members. The community awareness surveys will be conducted among community members to determine their level of awareness of the MDA campaign following mobilization activities in targeted communities (Table 2).

**Table 2:** Qualitative sample size and participant eligibility for the ETAS study

Thematic Areas	Aim	Sample size	Eligibility
Pre-MDA FGDs with drug distributors and MDA field supervisors present at the MDA training	Assess the acceptability of the increased number of rounds targeted to children among MDA implementers, and to better understand perceived barrier to access or implementation	3 FGDs with a total of 17 participants	MDA drug distributors and MDA field supervisors who participated in the MDA implementation training
Post-MDA FGDs with participating drug distributors	Assess the feasibility of additional treatment rounds targeted to children amongst MDA implementers	4 FGDs with a total of 24 participants	MDA field supervisors and drug distributors that participated in the extended rounds of MDA
Post-MDA FGDs with participating community members	Assess the acceptability of additional rounds of targeted MDA amongst the community members	8 FGDs with a total of 48 participants	Chiefs, mothers, and guardians/caretakers, regardless of whether their child has participated in the MDA or not
Community awareness survey	To assess the community’s knowledge, and awareness of MDA mobilization activities, and the role of health workers in mobilization for the MDA campaign. To evaluate the impact of communication and messaging to raise awareness of the MDA campaign	6 to 9 communities with a total of 120 to 180 participants	Community members of communities targeted for MDA

*FGD* Focus Group Discussion, *MDA* Mass Drug Administration

Mothers, caretakers, and chiefs from communities receiving enhanced interventions will be recruited for FGDs using purposive sampling. This approach ensures that participants are from different communities and have participated in the relevant activities that will be discussed during their FGD. One FGD will only include chiefs, because they have significant influence within the communities and other participants are unlikely to disagree with them publicly. Drug distributors from the county, recruited for the intervention, will also participate in the FGDs. Each FGD will be conducted for approximately 1.5 h by qualified research assistants who have received a 3-day training on research techniques. One moderator and 1 assistant will be assigned per FGD and will follow a comprehensive interview guide on key themes about the research question. All FGDs with community members and chiefs will be conducted in the local Toposa language. Assistants will audio record the discussions, verbally translate and record the Toposa audio into English, and then transcribe the English audio version. FGDs with MDA field supervisors will be conducted in English. FGD assistants will closely observe participants, their significant interactions, and the dynamics within the group, taking detailed notes on collective themes.

A community survey will be completed following MDA mobilization activities to gauge the community’s knowledge and awareness of the upcoming MDA campaign. The survey will be conducted at 3 time points. The first will be conducted before the first round of MDA in each study arm. Two communities will be randomly selected from each study arm for the survey. The census data from that community (previously collected) will be used to randomly select 20 households to survey. One person from each household will be interviewed for the survey by trained interviewers, with preference given for the female caregiver. The survey will be conducted a second time prior to the child-only treatment rounds in the thrice-treatment study arm, and then the survey will be conducted a third time in the bi-annual study arm prior to the second round of MDA.

### Surveys and trachoma monitoring

Household data collection and trachoma monitoring will take place through population-based surveys at baseline (prior to interventions) and approximately 12 months later. Sample size calculations will assume a TF prevalence of 4% among children ages 1 to 9 years with a precision of 2%, an assumed design effect of 3.0, and a 95% confidence level, for a total sample size of 1,104 children. Adjusting for an assumed non-response rate of 20% yields a population size of 1,325 children targeted for surveying. A multi-level cluster random sampling procedure will be used whereby 30 communities will be selected via simple random sample from a list provided by the South Sudan MOH Trachoma Control Program. We will employ the random sort technique to ensure an equal probability of selection across communities in the sampling frame. An independent sampling statistician will generate random values in SAS 9.4 (SAS Institute Inc.) with the ranuni function and a seed to allow replication of the sample should there be a need to augment the cluster list with additional clusters resulting from shortages in availability due to unforeseen problems in access or eligibility. In the second stage 6 segments of 5 households will be randomly selected through community mapping and segmentation for a total of 30 households in each of the 30 communities. Given an estimated 3.7 people per household, of which 40% are children, we would expect to survey on average 45 children ages 6 months to 9 years present per community. Therefore, for trachoma outcomes, we anticipate examining a total of 1,350 children ages 6 months to 9 years. A new random sample of 30 households from each of the same 30 study communities will be selected for the 12-month impact survey, plus 4 additional communities will be randomly selected from the county for inclusion to monitor trends in trachoma outcomes under standard-of-care, annual MDA, and to inform sample size calculations for a potential future efficacy trial. At each survey, a household questionnaire will collect demographic, socioeconomic, and water, sanitation, and hygiene (WASH) data using standard questions found in trachoma impact surveys [7].

Trained and certified trachoma graders will examine all individuals according to the WHO simplified grading system for trachoma using a 2.5 × magnification loupe and adequate light [30]. Tarsal conjunctivae photographs will be taken of both eyes of each child ages 6 months to 9 years using a cellscope device, a smartphone attachment that illuminates and magnifies the conjunctivae [31]. Photographs will be graded for the signs of trachoma. Conjunctival swabs to detect *Chlamydia trachomatis* (*Ct*) infection will be collected from children ages 6 months to 9 years whereby a gloved grader will firmly draw a swab 3 times over the left conjunctivae, rotating at each pass. Conjunctival swabs will be tested for infection and infectious load using polymerase chain reaction [32]. With the availability of additional funding, a sample of positive conjunctival swabs from each timepoint will be tested for antimicrobial resistance markers within the conjunctival space using genomic sequencing techniques.

### Data collection and management

A custom-built Android-based electronic data capture system (Conexus, Los Gatos, CA, USA) will be used for the census, data collection, and treatment phases of the study. This data is stored in a NoSQL database in Salesforce (Salesforce, San Francisco, CA, USA). Study participants will be assigned unique identification numbers to be used to link the census and treatment data and will not contain personal identifying information. Survey data will be collected using Open Data Kit (ODK) Collect to submit XML forms to NEMO, a form design and aggregation software built from the ODK software development kit. Qualitative data will be audio recorded, and some written notes may be recorded by facilitators. At the end of each day, or as frequently as possible, data collection devices will be connected to the internet so that data can be uploaded to the study server. All computers and other electronic devices will be password protected. An independent data monitoring committee will not be formed for this study, however, data from all phases of the study will be reviewed by data management staff to ensure quality. Upon completion of the study, de-identified data will be made available upon request of the South Sudan MOH.

### Ethics approval and consent to participate

This study has been approved by the South Sudan MOH Research Ethics Review Board at the Directorate of Policy, Planning, Budgeting, and Research; and the IRB of Emory University (00002467). Because of the low level of literacy within the population, verbal informed consent and assent will be sought from the community leader and each individual prior to distribution of study medications. Before the census or surveys are conducted in a community, oral informed consent will be obtained, first from the community leader, then from the adult household representative of those houses included in the study, and then from each participating individual. Within a household, field staff will fully explain the study purpose and what is included in the study. Field staff will explain that data collection tools will not seek sensitive or private information and that respondents will be free to terminate the interview at any time without need of explanation. Declining to participate in the survey or questionnaire will not influence their ability to receive any treatment indicated in the survey. The oral informed consent form script will be read to the participants prior to data collection. Oral consent will be recorded electronically. Major changes to the protocol will be reported to the Review Boards at Emory University and in South Sudan.

Risks and benefits of the study will be communicated to study staff and communities through community meetings. The medications to be administered are safe and used routinely by the South Sudan MOH and have been found to be safe. The trachoma grading is non-invasive, requiring only a visual inspection. Collection of ocular swabs for detection of trachoma infection is associated with minor discomfort due to the swab abrading the conjunctiva. Qualitative data collection will not ask questions that cover invasive or sensitive issues. There are unavoidable opportunity costs, though screening all the members of a household and conducting the study data collection should take less than 30 min. The physical, social, or psychological risk associated with participation in this survey is no more than the risk associated with daily life. There are no economic risks associated with this study. All individuals with signs of trachoma will be provided TEO, and individuals identified with TT, the blinding stage of trachoma, will be registered and informed when free eyelid surgery will be provided in their county. There will be additional indirect community benefits in that the data from the study may inform decisions about future form and delivery of the trachoma control programs. Compensation will be provided to FGD participants to reflect the extended time devoted to participating in the discussion.

### Dissemination plan

Reports detailing study findings will be shared firstly with the South Sudan MOH and local health departments. Information gained from the study will also be shared with communities during MDA trainings and post-MDA review meetings. The results will further be shared with local partner organizations, as well as international organizations through program reviews, meetings, scientific conferences, and peer-reviewed journals. Study data will also inform future trachoma-specific intervention effectiveness trials in South Sudan and other countries seeking to accelerate progress towards eliminating trachoma as a public health problem. De-identified trial data will be made publicly available upon request of the South Sudan MOH after publication of primary outcomes.

## Discussion

The results of this study will provide key information to trachoma programs on whether enhanced antibiotic interventions are affordable, sustainable, and acceptable to communities. Recently it has been recognized that some countries are taking much longer than expected to reach trachoma elimination thresholds with standard SAFE interventions [2, 33]. As a result, there has been growing interest in intensifying the antibiotic component of SAFE, with WHO convening an informal consultation meeting with experts in December 2021 to discuss possible strategies. While the ability to intensify antibiotic treatments represents an opportunity for highly trachoma endemic countries to reach elimination faster, there is still much for programs to consider before enhancing MDA interventions. Published data on the costs of implementing trachoma MDA within South Sudan are rare and need to be updated to understand the implications of transitioning to more intense strategies [34]. Further, ensuring high MDA coverage under new treatment regimens will require the support and acceptance of both the drug distributors and the communities they serve. This study has received ethical clearance and is expected to be conducted between 2022 and 2023. The data generated from this study, coupled with comparative efficacy data from other potential future randomized trials, could lead to policy changes around the mass delivery of antibiotics in countries with a high burden of trachoma.

This study will utilize two MDA strategies that represent enhancements to the standard-of-care annual MDA. In a previous 3-country study, it was demonstrated that a 3-dose strategy could drastically reduce ocular *Ct* infection among children [11]. Treating communities with multiple rounds within a short period of time could be programmatically favorable as it would allow for a single drug shipment and a single training prior to intervention. Targeting children may also be more cost effective as it requires less medication than a community-wide MDA and yet targets the age group shown to act as an infection reservoir within communities [9, 11, 32]. Recently a modeling study demonstrated the efficacy of this 3-dose, targeted strategy on *Ct* infection, however, it has yet to be implemented within actual communities. The second enhanced MDA strategy in this study, biannual MDA, has been compared to annual MDA in several randomized trials conducted in Ethiopia [35, 36]. Although biannual MDA has not shown to be statistically significantly more effective than standard-of-care annual MDA in these past trials, a faster time to elimination in some communities was observed. Examining the cost and community acceptability of these enhanced MDA strategies in a real-world setting will provide valuable data to better understand the programmatic implications of increasing the frequency and targeting of MDA interventions.

Potential limitations of our study design include generalizability and adequate statistical power for comparing trachoma outcomes. This study will take place within a county in South Sudan which has a mature MDA program; therefore, results may not be generalizable to counties introducing MDA for the first time. Since study communities have been receiving annual MDA for 5 years or more, they may be more accepting of more intense and targeted MDA strategies then treatment-naïve communities. This study was not designed as a clinical trial to compare the efficacy of enhanced antibiotic treatments on trachoma outcomes, instead the aim was to determine the cost and community acceptability of 2 enhanced treatment strategies. Given that the counties in Eastern Equatoria state have received 5 or more rounds of MDA prior to the start of this study, it is likely that the prevalence of *Ct* infection would be too low to allow for detection of a statistically significant difference between treatment arms at the end of this study [7]. Despite this, the trachoma-specific data generated from this study will be used for programmatic monitoring and to inform appropriately powered efficacy trials within South Sudan. With any large-scale antibiotic intervention, there is a concern of antimicrobial resistance within the recipient population. While these intense strategies provide more antibiotic up front, the assumption is that they will allow the program to cease distribution of antibiotic sooner than the current expected timeline, and therefore could potentially save years of antibiotic distribution overall. Lastly, while this study focused on the A component of SAFE, certainly more studies on the costs and feasibility of various F and E interventions in settings such as South Sudan are needed.

Enhanced MDA approaches could be 1 method for South Sudan and other highly endemic countries to reach elimination thresholds by 2030 and could further help countries recover from programmatic delays caused by conflict or humanitarian emergencies. The ETAS study will provide trachoma control programs with valuable insight on the affordability and feasibility of enhanced MDA interventions for the global goal of eliminating trachoma as a public health problem.

## Supplementary Information

**Additional file 1: Supplemental Table S1.** Tracing factors used in the ETAS study. This table describes each cost category and provides information on how those costs are split divided into the implementation setting (research activities excluded), and costs related to the study setting (including study and implementation costs).

## Data Availability

Not applicable.

## Abbreviations

*Ct*: *Chlamydia trachomatis*
ETAS: Enhancing the A in SAFE
FGD: Focus group discussion
GET2020: Alliance for the Global Elimination of Trachoma by the year 2020
GPS: Global positioning system
IRB: Institutional Review Board
ITI: International Trachoma Initiative
MDA: Mass drug administration
MOH: Ministry of Health
NTD: Neglected tropical disease
ODK: Open Data Kit
POS: Powder for oral suspension
SAE: Severe adverse event
SAFE: Surgery, antibiotics, facial cleanliness, and environmental improvement
TEO: Tetracycline eye ointment
TF: Trachomatous inflammation-follicular
TT: Trachomatous trichiasis
WASH: Water, sanitation, and hygiene
WHO: World Health Organization
