# Supplementary material for: Cost and community acceptability of enhanced antibiotic distribution approaches for trachoma in the Republic of South Sudan: enhancing the A in SAFE (ETAS) study protocol
Source: BMC Ophthalmol. 2023 Feb 6;23:51. doi: 10.1186/s12886-023-02783-x (PMC9900535; doi:10.1186/s12886-023-02783-x)
Supplement: Supplementary file 1 — Additional file 1: Supplemental Table S1. Tracing factors used in the ETAS study. This table describes each cost category and provides information on how those costs are split divided into the implementation setting (research activities excluded), and costs related to the study setting (including study and implementation costs). [file 12886_2023_2783_MOESM1_ESM.docx]

Supplemental Table S2. Tracing factors used in the ETAS study

| Line item | Expenditure time | Type of shared costs | Tracing factors*: Total to study portion | Tracing factors: Study portion to activities |
| --- | --- | --- | --- | --- |
| Staff time | Salaries of health and other staff, including international staff and Ministry of Health staff | Allocation to study activities | % of time | % of time |
| Other staff | Cost of community health workers and volunteers | Allocation to study activities | % of time spent on study activities as recorded during data collection | Allocate to the activities most relevant such as outreach or social mobilization (% of time) |
| Drug | Cost of drugs shipped | NA | 100% to MDA | NA |
| Equipment | Annualized capital cost of equipment | • Office equipment to wide range of possible activities | • % used for MDA (proportion of space use for routine) • Focus on equipment that is used for routine MDA, such as a computer used by the EPI manager.  Include all specialist software required. | • Allocate equipment to program management activity |
| Vehicles | Annualized capital cost of vehicles | • Vehicles at facility to mainly outreach and drug collection • At higher levels to management/ surveillance etc | Daily cost of vehicle use. | If the vehicle logbook contains details on the purpose of the trip, then use these details to allocate. Otherwise, estimate ratios from the number of trips and kms per trips over total kms travelled for activities. |
| Building | Building or rental value | Health posts/other fixed sites | Number of square meters for the area relevant for routine MDA (where drug is administered, stored), or % of facility footprint allocated to MDA | Allocate 100% of health posts to outreach (if applicable) |
| Transport | Specific questions for transport for: • Outreach • Drug collection • Supervision • Meetings (social mobilization) • Other | • All allocated directly to correct activity • If something crops up under Other, we will have to choose the most likely fit (e.g., surveillance) | • 100% to MDA, unless trips are multi- purpose.  • If unable to allocate directly, use the same ratios as generated for vehicles | • 100% to best fit activity • For a trip that involved more than one purpose, allocate evenly across purposes |
| Training | All training-related costs including per diems, printing, and travel allowances | NA | 100% to MDA | 100% to training |
| Social mobilization | Per diems | Any transport costs to be recorded under transport tab | 100% to MDA | 100% to social mobilization |
| Cold chain operating and maintenance | Various fuels and maintenance | Energy consumption for the facility needs to be allocated to routine MDA | • Maintenance share for MDA asked directly in the questionnaire • Cold chain energy costs best based on kw/hour and the unit cost/kw hour | • 100% to cold chain maintenance • Estimated cold chain energy cost should not be double-counted in Overhead cost below |
| Overhead costs | Expenditures for heating, phone, internet, electricity, and stationery | Need to allocate first to routine MDA and then to activities | Total routine doses/number of outpatient visits | Allocate all to program management (costs should be net of cold chain energy) |
| Health committee meetings and stakeholder groups | Mainly qualitative questions | The value of community participant time should be costed | The value of community participant time should be costed | 100% to social mobilization |

ETAS-Enhancing the A in SAFE; MDA= Mass Drug Administration.

* Determining the proportion of a shared resource to allocate to MDA to estimate MDA-specific costs can be difficult. One approach is to allocate costs based on an “allocation key” or “tracing factor.” The tracing factors described below can also be used to allocate input costs within MDA to different program activities. Supplementary table 1 provides examples of the use of tracing factors.
